# Tobacco Smoking or Nicotine Phenotype and Severity of Clinical Presentation at the Emergency Department (SMOPHED): Protocol for a Noninterventional Observational Study

**DOI:** 10.2196/54041

**Published:** 2024-04-24

**Authors:** Davide Campagna, Konstantinos Farsalinos, Giorgio Costantino, Giuseppe Carpinteri, Pasquale Caponnetto, Francesca Cucuzza, Riccardo Polosa, SMOPHED Study Group

**Affiliations:** 1 Department of Clinical and Experimental Medicine University of Catania Catania Italy; 2 Center of Excellence for the Acceleration of Harm Reduction University of Catania Catania Italy; 3 Emergency Department Policlinico Teaching Hospital Catania Italy; 4 Department of Public and Community Health School of Public Health University of West Attica Athens Greece; 5 Scientific Institute for Research, Hospitalization and Healthcare Ca' Granda Ospedale Maggiore Policlinico Unità Operativa Complessa Pronto Soccorso e Medicina d'Urgenza University of Milan Milan Italy; 6 Department of Educational Sciences Section of Psychology University of Catania Catania Italy; 7 See Acknowledgments

**Keywords:** NEWS, National Early Warning Score, emergency department, smoking, nicotine/tobacco use, electronic cigarettes, heated tobacco products

## Abstract

**Background:**

In the last few years, several nicotine products have become available as alternatives to smoking tobacco. While laboratory and limited clinical studies suggest that these devices are less toxic compared to classic tobacco cigarettes, very little is known about their epidemiological impact. Visiting the emergency department (ED) often represents the first or even the only contact of patients with the health care system. Therefore, a study conducted at the ED to assess the impact of these products on health can be reliable and reflect a real-life setting.

**Objective:**

The aim of this noninterventional observational study (SMOPHED study) is to analyze the association between the severity of clinical presentation observed during ED visits among patients using various nicotine products and the subsequent outcomes, specifically hospitalization and mortality.

**Methods:**

Outcomes (hospitalization and mortality in the ED) will be examined in relation to various patterns of nicotine products use. We plan to enroll approximately 2000 participants during triage at the ED. These individuals will be characterized based on their patterns of tobacco and nicotine consumption, identified through a specific questionnaire. This categorization will allow for a detailed analysis of how different usage patterns of nicotine products correlate with the clinical diagnosis made during the ED visits and the consequent outcomes.

**Results:**

Enrollment into the study started in March 2024. We enrolled a total of 901 participants in 1 month (approximately 300 potential participants did not provide the informed consent to participate). The data will be analyzed by a statistician as soon as the database is completed. Full data will be published by December 2024.

**Conclusions:**

There is substantial debate about the harm reduction potential of alternative nicotine products in terms of their smoking-cessation and risk-reduction potential. This study represents an opportunity to document epidemiological data on the link between the use of different types of nicotine products and disease diagnosis and severity during an ED visit, and thus evaluate the harm reduction potential claims for these products.

**International Registered Report Identifier (IRRID):**

PRR1-10.2196/54041

## Introduction

Smoking-related diseases are a well-known group of pathologies that are responsible for 8 million deaths per year globally [1]. Despite numerous restrictions, taxation, and communication campaigns, there are still more than 1.3 billion smokers globally. Quitting smoking remains one of the most cost-effective methods to reduce health risks. However, the success rate of smoking cessation methods remains low in real-world settings [2-5]. Additionally, a substantial proportion of smokers are unwilling to use smoking cessation services but would prefer switching to a less harmful alternative product [6,7]. In the last few years, several alternatives to smoking nicotine products have become available, which are commonly referred to as electronic nicotine delivery systems (ENDS), namely e-cigarettes and heated tobacco products (HTPs). While laboratory and limited clinical studies suggest that these products reduce exposure to toxins and may thus reduce health risks [8-12], very little is known about their epidemiological impact.

Several studies have demonstrated a high proportional cigarette smoking prevalence in patients presenting to the emergency department (ED), which is higher than the prevalence in the general population [13-19]. This is expected considering the disease burden caused by smoking, which might result in more ED visits for smokers compared to nonsmokers. Current smoking as well as smoking relapse were found to be significantly associated with ED visits [20-24]. Access to the ED often reflects the first or the only contact of patients with the health care system; thus, the ED visit represents an opportunity to record smoking and nicotine use patterns. However, no studies have systematically recorded use patterns for ENDS in relation to ED visits. Considering the growing popularity of these products and the limited epidemiological evidence on their health effects, a detailed recording of use patterns for all nicotine products is important to monitor their use prevalence, health care system burden, and epidemiological impact.

The National Early Warning Score (NEWS) is a standardized tool used for assessing and responding to acute illness in patients admitted to the ED [24]. The NEWS is based on a straightforward aggregate scoring system, assigning scores to routine physiological measurements taken when patients either present at or are monitored in the hospital. The scoring system is based on six key physiological parameters: (1) respiration rate, (2) oxygen saturation, (3) systolic blood pressure, (4) pulse rate, (5) level of consciousness or new confusion (alert, verbal, pain, unresponsive [AVPU] scale), and (6) temperature.

For patients accessing the ED, the NEWS aids triage nurses in determining the appropriate urgency level for patient examination, which is represented by color codes. This stratification is based on the severity and urgency of the patient’s condition.

The aim of this study is to accurately document the patterns of smoking and nicotine product use, including ENDS, in patients visiting the ED. A key focus is to investigate whether the use of ENDS is associated with any measurable differences in disease severity and outcomes compared to conventional tobacco smoking among these patients.

## Methods

### Study Design

The Smoking or Nicotine Phenotype and Severity of Clinical Presentation at the Emergency Department (SMOPHED) study is an observational study with no intervention or randomization, analyzing the association between the patient’s health condition during an ED visit as well as the outcome (hospitalization and death) and different patterns of nicotine products use. Specifically, the study will explore relevant associations according to the smoking and ENDS product use status (current, former, and never use). The pilot phase of the study will take place at a single clinical center, aiming to evaluate its feasibility and analyze the results. Upon successful completion, there are plans to expand the study into a multicenter format involving a national network comprising 10-12 EDs.

### Study Population

Inclusion and exclusion criteria are summarized in Textbox 1. Participants will be recruited among patients presenting at the ED of Policlinico Teaching Hospital of Catania, Italy, in 1 month during the diurnal shift. Only patients that access the ED for a nontraumatic reason will be screened, since it is unlikely that smoking or use of other nicotine products can have any causal effect on accident incidence and subsequent diagnosis. The study will take place during daytime working hours owing to organizational constraints. Typically, our ED admits approximately 70-80 patients for nontraumatic issues during a daytime shift, which runs from 8 AM to 8 PM.

We calculated the expected sample size for the study based on available national and regional statistics, providing a practical approach to estimating the study’s sample size. The current and former smoking rates in Sicily are estimated at 22.5% and 13.3%, respectively [25], while the combined prevalence of combustion-free products use (e-cigarettes and HTPs) stands at approximately 5% [26]. Considering the annual Catania ED access rate of 80,000 (monthly access rates of 6000-6500) [27], we anticipate recruiting no less than 1500 current smokers and 1000 former smokers per month. Additionally, we expect to enroll no less than 350 users of combined e-cigarettes/HTPs per month (dual usage is estimated at approximately 50% of the total). Since approximately 70 patients per day access the ED during the diurnal shift for nontraumatic reasons, we expect to enroll approximately 2000 participants in 1 month.

**Inclusion and exclusion criteria for study participation.**

Inclusion criteria

- Adult (aged≥18 years)
- Ability to understand and sign the informed consent form

Exclusion criteria

- Accessed the emergency department for a traumatic accident
- Pregnancy

### Ethical Considerations

The study will be conducted according to the principles of Good Clinical Practice and the Declaration of Helsinki. Ethical clearance for this protocol was obtained through the ethical review board “Comitato Etico Catania 1” at Policlinico Teaching Hospital of Catania (84/2023/PO del Registro dei pareri del CE) on April 17, 2023. If any amendments to this protocol are required, the chief investigator will be responsible for the decision to amend the protocol and for deciding whether an amendment is substantial or nonsubstantial. Any substantial amendments will be submitted to the research ethics committee for approval before implementation. Informed consent from study participants will be obtained by the investigators through relevant forms. A dedicated website will be created for the study. This website will respect the General Data Protection Regulation policy. Every study team member will access the site with a personal and unique ID and password. The website will create two different databases to guarantee participants’ privacy. The first database will record the fiscal code of the participant and will assign a unique ID. The second database will anonymously record the data from each participant using only the participant’s ID. It is necessary to create two databases so that additional information on outcomes or other missing data will be recorded at a later time.

Patients will be asked for authorization to use their clinical data in an anonymous format, in accordance with the current privacy directives related to access to the ED. The informed consent model will be shown to participants who must be able to understand and sign it as an inclusion criterion to participate in the study. All collected informed consent forms will be stored at the ED of Policlinico Teaching Hospital of Catania. No financial or other incentives will be provided to the participants.

### Data Collection

On arrival at the ED, each patient undergoes a triage phase. A nurse is usually responsible for evaluating the severity of the health condition and the priority for clinical assessment (unless the patient enters the ED by ambulance under a red code). The nurse usually performs this task by collecting vital signs and recording the patient’s medical history. This information is then used to assign a color code representing the priority for further assessment. The patient then waits for the medical evaluation in a dedicated room where nurses can monitor the clinical condition of all attending patients. Participants who meet the inclusion criteria will be recruited during this phase and will be administered an electronic questionnaire (see Multimedia Appendix 1) about their smoking and nicotine product use habits and their medical history. This questionnaire serves to typify the patient’s use phenotype and quantify exposure to each product.

In addition to the questionnaire, medical data recorded at the triage phase, such as the NEWS, heart rate, blood pressure, blood oxygen saturation, respiratory rate, temperature, and fraction of inspired oxygen, will be collected.

After the assessment and management of each patient in the ED, the final diagnosis, final disposition, and color code at discharge or admission to a ward will be recorded (see Table 1). To avoid compromising the health of patients with severe disease requiring urgent medical attention, potential participants who arrive at the ED under a red code (ie, with a severe acute illness) will be asked to participate only after they are stabilized (ie, the questionnaire, NEWS, and vital signs will be recorded later).

Based on the responses to the questionnaire, participants will be divided in the following 5 smoking subgroups to facilitate analysis: (1) current tobacco smoker, (2) former tobacco smoker, (3) former tobacco smoker now using e-cigarettes and HTPs (exclusive use), (4) current tobacco smoker also using e-cigarettes and HTPs (dual use), and (5) never smokers or e-cigarette/HTP users.

After 1 month, data in the electronic case report form (eCRF) will be extracted for the statistical analysis.

Since the ED is usually very crowded, four study team personnel will work on the study and will screen and recruit patients during the waiting period before the medical examination. The study team will interview potential participants in a dedicated room within the ED area to guarantee privacy. The initial survey will last approximately 5 minutes per patient. This is a reasonable time to collect product use information from each participant. After the medical visit and final disposition, compilation of the eCRF will be completed as provided by the study protocol, with each study member collecting all necessary information about the patients’ outcome (discharge or admission to a ward) by the end of their shift. Some patients may stay in the ED beyond the length of a diurnal shift; in such a case, the data will be collected the day after their arrival. Some patients might access the ED two or more times during the study. To avoid double entries, only the first visit will be included for analysis in the study. The eCRF database will reply with an error if we try to recruit a patient with the same fiscal code a second time (a fiscal code is a unique identifying code in Italy, and it is mandatory to be recorded for each patient during the triage phase in the ED).

**Table 1:** Data to record in the electronic case report form.

Data type			Value/units
NEWS^a^ calculated at the patient’s triage phase (conventionally, patients with an acute condition will be recorded as being at high risk, corresponding to NEWS>7)			0-17
**Vital signs recorded during triage**			
	Heart rate	Beats per minute	
	Blood pressure	mmHg	
	Blood oxygen saturation	%	
	Respiratory rate	Breaths per minute	
	Body temperature	°C	
	Fraction of inspired oxygen	%	
Smoking questionnaire			See Multimedia Appendix 1
Medical history and drug use history			Textual
Length of stay in the ED^b^ or in the hospital (if admitted))			Hours and minutes, or days if admitted
Final diagnosis of discharged patients			Textual
Final disposition			Admitted/discharged
Final diagnosis of admitted patients			*ICD-9*^c^ codes

^a^NEWS: National Early Warning Score.

^b^ED: emergency department.

^c^*ICD-9: International Classification of Diseases, 9th edition*.

### Endpoint and Outcomes

The primary endpoint of the study is the association between the NEWS and product use phenotypes. Our hypothesis is that use of ENDS may be associated with a lower NEWS compared to cigarette smoking. If our hypothesis will be confirmed, the study will be replicated as a multicenter study to validate our findings.

Secondary outcomes will be hospital admissions (vs discharge) and length of stay in the ED and in the hospital (if admitted). Moreover, we will compare the prevalence of acute diseases known to be related to smoking between groups, specifically stroke, acute myocardial infarction, peripheral artery diseases, chronic obstructive pulmonary disease, asthma, and respiratory infections.

### Statistical Analysis

Descriptive analysis will be performed by presenting numerical data as mean (SD) and categorical data as n (%). Patients will be classified according to product use as a current tobacco smoker, former tobacco smoker, former tobacco smoker now using e-cigarettes and HTPs (exclusive use), current tobacco smoker also using e-cigarettes and HTPs (dual use), and never smokers/never e-cigarette or HTP users.

Univariate comparisons will be performed using *χ*^2^ tests for categorical variables and the Kruskal-Wallis H test for the NEWS. Regression analyses will be performed to examine the association between the use of different types of products and the NEWS as well as secondary outcomes. Demographics, including age, sex (male or female), and educational level, as well as past medical history will be included as independent variables. Since the majority of ENDS users report current or past smoking, the smoking status of these users will also be recorded along with those who do not report any ENDS use. Therefore, the analysis will be adjusted for the smoking status of ENDS users. Additionally, secondary analyses will be performed for never-smoking ENDS users, if a sufficient number of such participants will be available. All analyses will be performed using SPSS v.25 (IBM) and a *P* value <.05 will be considered statistically significant.

### Dissemination

The intention of the authors is to disseminate the results of the study through articles in high-quality, peer-reviewed journals and through conference abstracts.

## Results

In March 2024, we have enrolled a total of 901 participants (approximately 300 potential participants did not provide the informed consent to participate). The data will be analyzed by a statistician as soon as the database is completed. Full data will be published by December 2024.

## Discussion

This protocol was designed to evaluate the impact of smoking and the use of other nicotine products on ED visits, including the respective clinical diagnoses and outcomes. Smoking is a leading preventable cause of morbidity and mortality. It is estimated that tobacco use accounts for 3%-6% of all ED visits and 5%-16% of total hospital expenses [28-30]. Smokers with preexisting diseases such as diabetes and asthma are more prone to ED visits than nonsmokers with similar conditions [23,31].

The smoking prevalence among patients at the ED surpasses that of the general population [16,18], indicating the ED as a key setting for managing smoking-related health issues. In recent years, there has been an intense debate about the harm reduction potential of ENDS. Some studies have shown their effectiveness in smoking cessation [32], yet there is scarce epidemiological evidence on the clinical impacts of noncombustible nicotine products. This study will be the first to explore the link between the use of various nicotine products and ED visits, as well as their association with urgent care needs for smoking-related disease.

Approximately one decade ago, our research group conducted the ECLAT study, which was the first randomized controlled trial that established the efficacy and the safety of e-cigarettes as smoking substitutes [33]. Since then, a newer generation of e-cigarettes, which are more efficient in nicotine delivery and more appealing, have shown encouraging results in clinical and real-world settings [34-41]. Therefore, they may represent a viable alternative for individuals unable or unwilling to quit smoking using approved methods. Nonetheless, it is important to thoroughly investigate and document the potential risk-reduction potential of these products in a clinical context.

Little epidemiological evidence exists on the harm reduction potential of ENDS. This study thus represents an opportunity to document epidemiological data on the link between the use of different nicotine products and disease severity during an ED visit, as well as clinical outcomes and associations with specific (ie, smoking-related) diseases. Besides further exploring the epidemiological impact of ENDS on diseases related to smoking, the implications of this study are also directly related to understanding the currently unknown burden of these products on emergency care resource use, particularly in the context of the well-known substantial effects of smoking on increasing ED visits. The systematic tracking of the impacts of nicotine use in this study will help to highlight the magnitude of tobacco and nicotine use in the community in relation to access to emergency health care services, and will enable those responsible for tobacco control policies, programs, research, and surveillance to assess the situation and inform decision-making.

This study, currently conducted in a single hospital, serves as a pilot phase, aiming to evaluate its feasibility and analyze the results. The ultimate objective is to expand this research to additional EDs within a national network.

Documenting the association between ENDS use and ED visits will provide insights on both local and central policy levels, especially when we consider that emergency and urgent care systems struggle with major challenges in developed countries with crowding, long waiting times, and, in general, increasing numbers of ED visits [42]. Therefore, it is important to examine factors associated with ED visits and adverse outcomes, and to assess if ENDS affect the ED burden in a different manner to smoking. At the same time, this study may document the need for routinely recording nicotine use habits for all patients accessing the ED instead of the current norm of recording smoking status only.

This study has several limitations. The brief period of recruitment and the use of only one study site may introduce sampling bias due to seasonality and locality. Therefore, this study may have limited generalizability to all Italian EDs. However, as mentioned above, this represents a pilot study with the purpose of further expanding the protocol to more regions and several EDs from other hospitals. While it is possible to verify exposure to tobacco smoke by measuring exhaled carbon monoxide and exposure to nicotine by measuring salivary cotinine, logistical and financial restrictions within the short duration of an ED presentation, as well as the urgent nature of attendance requested in the ED, preclude us from performing an objective assessment of the smoking status. Although self-report of smoking status has been shown to be accurate in the ED setting [17], a systematic review (despite including studies outside the ED setting) showed some discrepancy between self-report and objective assessment [43]. In addition, determining nicotine exposure would not help to distinguish between the use of tobacco cigarettes and noncombustible nicotine products. Moreover, the responses to questions on past smoking and nicotine use habits may be introducing recall bias. Finally, the exclusion of patients in a critical state who need urgent care will be compensated by a subsequent request to participate in the study once they are stabilized.

In conclusion, this study protocol addresses the need for additional epidemiological evidence on the effects of ENDS on health, with clinical endpoints. The ED setting represents an opportunity to record smoking and nicotine habits, assess the burden of different products, and provide guidance for interventions at both the personal and systematic level.

## Appendix

Multimedia Appendix 1 Questionnaire to be administered to patients.

## Abbreviations

AVPU: alert, verbal, pain, unresponsive
eCRF: electronic case report form
ED: emergency department
ENDS: electronic nicotine delivery system
HTP: heated tobacco product
NEWS: National Early Warning Score
SMOPHED: Smoking or Nicotine Phenotype and Severity of Clinical Presentation at the Emergency Department
