# Supplementary material for: Tobacco Smoking or Nicotine Phenotype and Severity of Clinical Presentation at the Emergency Department (SMOPHED): Protocol for a Noninterventional Observational Study
Source: JMIR Res Protoc. 2024 Apr 24;13:e54041. doi: 10.2196/54041 (PMC11079756; doi:10.2196/54041)
Supplement: Multimedia Appendix 1 [file resprot_v13i1e54041_app1.docx]

**QUESTIONNAIRE-Smoking & nicotine use**

**DEMOGRAPHICS**

1. **Age**
2. **Gender**
3. **Males**
4. **Females**
5. **What is your highest education level (please adjust according to the educational system in Italy)**
6. **High school or less**
7. **Technical education**
8. **University education**
9. **Postgraduate education**
10. **Marital status**
11. **Single**
12. **Married/living with partner**
13. **Divorced/widowed**

**TOBACCO CIGARETTES**

1. Regarding tobacco cigarette smoking, you are:
2. Current smoker (you currently smoke ready-to-use cigarettes or roll-your-own or pipes or cigars or cigarillos) – ***GO TO Q2***
3. Former smoker (used to smoke but not now) – **GO TO Q5**
4. You have never smoked in your life – **GO TO Q10**
5. Do you currently smoke:
6. Daily – **GO TO Q3**
7. Occasionally (not every day) – **GO TO Q4**
8. How many cigarettes do you smoke daily?
9. For how long have you been smoking?

MONTHS
YEARS

- **GO TO Q10**

1. When you were a smoker, did you smoke:
2. Daily – **GO TO Q6**
3. Occasionally (not every day) – **GO TO Q7**
4. How many cigarettes did you smoke daily?
5. For how long had you been smoking?

MONTHS
YEARS

1. How long ago did you stop smoking (in days and/or months and/or years – please tick the corresponding option)?
2. YEARS
3. MONTHS
4. Days (if you have quit smoking for < 1 month)
5. How did you manage to quit smoking? (You can choose more than 1 answer if you used multiple aids)
6. By yourself
7. Using medicinal nicotine substitutes (stickers, chewing gum, sprays, etc.)
8. By using oral medications to quit smoking
9. Using psychological support
10. By using an electronic cigarette
11. Using a heated tobacco cigarette
12. Other (specify)

- **GO TO Q10**

**E-CIGARETTE USE**

1. Concerning electronic cigarettes (e-cigarettes), you:
2. Are using them NOW **– GO TO Q11**
3. You used them in the past, but not now **– GO TO Q15**
4. You have never used them **– GO TO Q20**
5. Do you currently use e-cigarettes:
6. Daily – **GO TO Q12**
7. Occasionally (not every day) – **GO TO Q13**
8. How many pods or ml of liquid do you consume daily?
9. Do you use nicotine in e-cigarettes
10. Yes
11. No
12. For how long have you been using e-cigarettes?

MONTHS
YEARS

- **GO TO Q20**

1. When you were using e-cigarettes, were you using them:
2. Daily – **GO TO Q16**
3. Occasionally (not every day) – **GO TO Q17**
4. How many pods or ml of liquid did you consume daily?
5. Did you use nicotine in e-cigarettes?
6. Yes
7. No
8. For how long had you been using e-cigarettes?

MONTHS
YEARS

1. How long ago did you stop using e-cigarettes (in days and/or months and/or years – please tick the corresponding option)?
2. YEARS
3. MONTHS
4. Days (if you have quit smoking for < 1 month)

- **GO TO Q20**

**HEATED TOBACCO PRODUCTS (IQOS, Glo, etc)**

1. Concerning heated tobacco products, you:
2. Are using them NOW **– GO TO Q21**
3. You used them in the past, but not now **– GO TO Q24**
4. You have never used them **– GO TO Q28 (END OF QUESTIONS ABOUT NICOTINE PRODUCTS)**
5. Do you currently use heated tobacco products:
6. Daily – **GO TO Q22**
7. Occasionally (not every day) – **GO TO Q23**
8. How many tobacco sticks do you consume daily?
9. For how long have you been using heated tobacco products?

MONTHS
YEARS

- **GO TO Q28 (END OF QUESTIONS ABOUT NICOTINE PRODUCTS)**

1. When you were using heated tobacco products, were you using them:
2. Daily – **GO TO Q25**
3. Occasionally (not every day) – **GO TO Q26**
4. How many tobacco sticks did you consume daily?
5. For how long had you been using heated tobacco products?

MONTHS
YEARS

1. How long ago did you stop using heated tobacco products (in days and/or months and/or years – please tick the corresponding option)?
   1. YEARS
   2. MONTHS
   3. Days (if you have quit smoking for < 1 month)

**(END OF QUESTIONS ABOUT NICOTINE PRODUCTS)**
